# Supplementary material for: Formation of Transient Protein Aggregate-like Centers Is a General Strategy Postponing Degradation of Misfolded Intermediates
Source: Int J Mol Sci. 2023 Jul 7;24(13):11202. doi: 10.3390/ijms241311202 (PMC10342647; doi:10.3390/ijms241311202)
Supplement: Supplementary file 1 [file ijms-24-11202-s001.zip › ijms-2478597-supplementary.pdf]

Supplementary Material

Formation of transient protein aggregate-like centers is a general strategy postponing degradation of misfolded intermediates.

Boronat et al.

Includes:

8 supplementary figures

2 supplementary tables

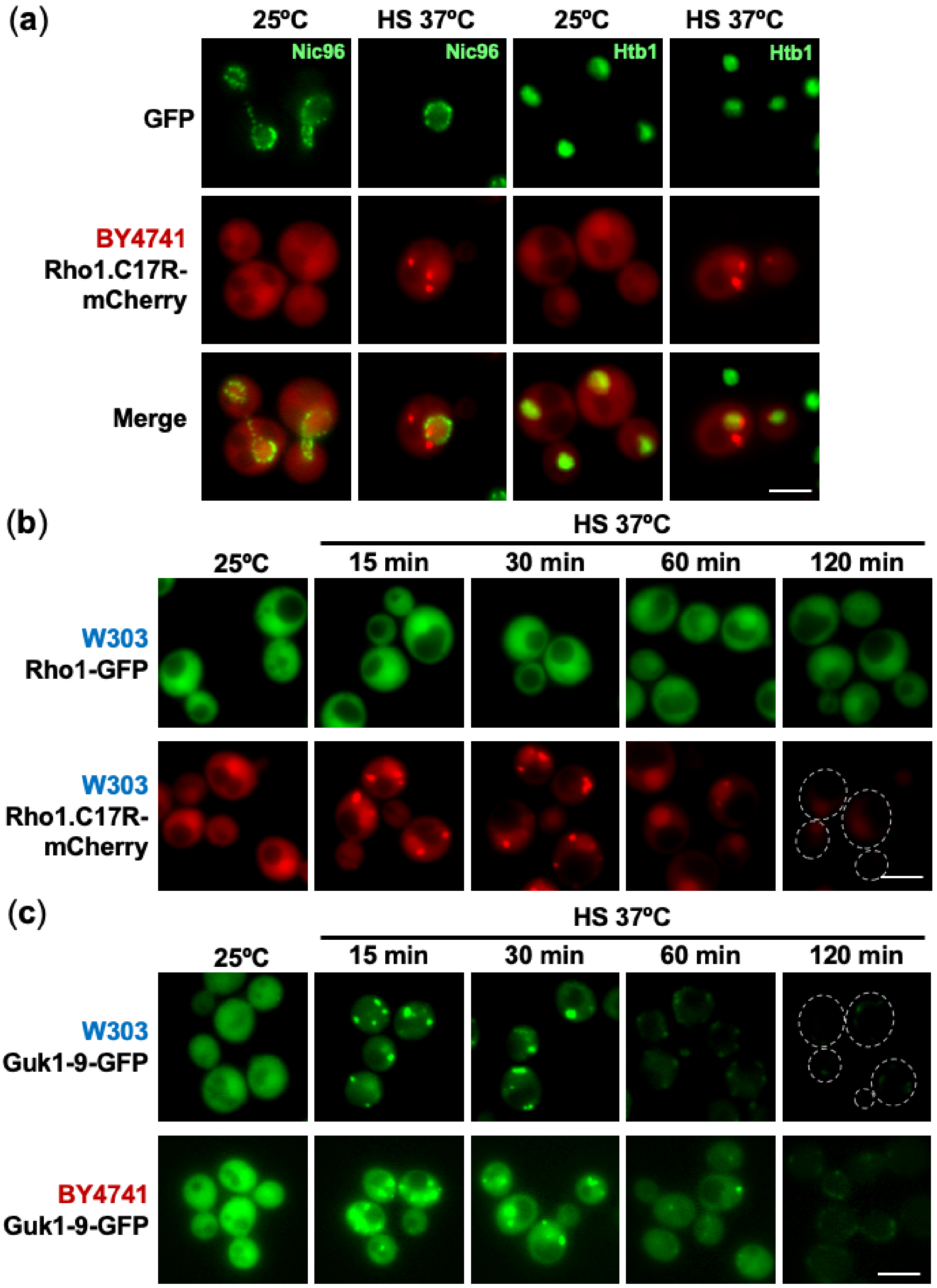

Figure S1. PAC formation is triggered by protein misfolding at non-permissive temperature. (**a**) Microscopy of cells co-expressing Rho1.C17R-mCherry and nuclear markers (Nic96 or Htb1-GFP) after heat-shock at 37ºC for 30 min. (**b**) Rho1 WT is not sequestered into PACs during heat shock. Cells carrying Rho1-GFP or Rho1.C17R-mCherry were incubated at 37ºC for the indicated time points and analyzed by fluorescence microscopy. (**c**) The misfolding reporter Guk1-9 is present at PACs upon mild temperature upshift. Distribution of Guk1-9-GFP during heat shock at 37ºC was examined by fluorescence microscopy in two budding yeasts strains, W303 and BY4741. Scale bar, 5 μm.

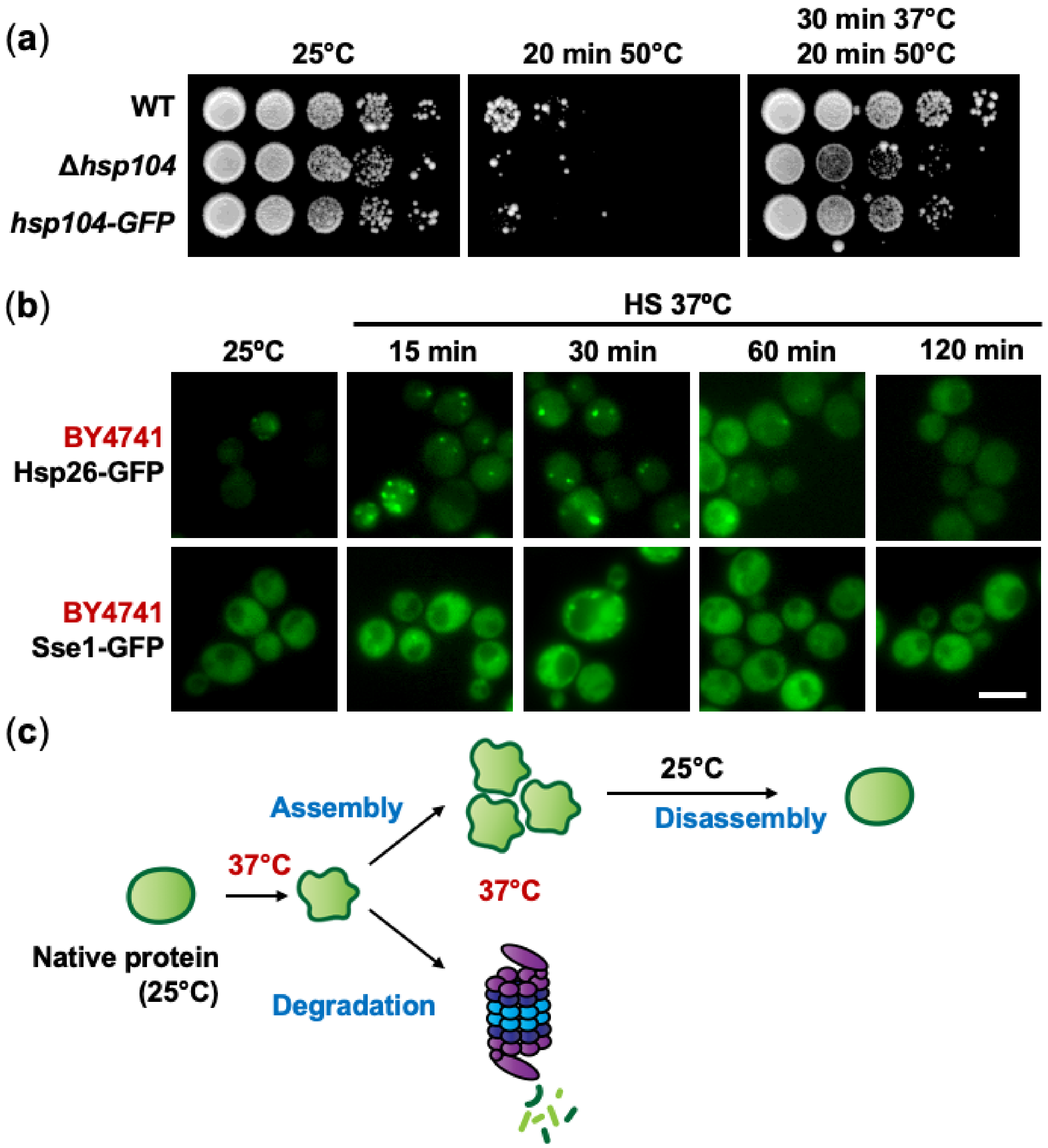

Figure S2. Study of functionality and localization of GFP-tagged chaperones. (**a**) The version Hsp104-GFP is not completely functional. WT, Δ*hsp104* and *hsp104-GFP* cells were incubated at different stress conditions (50ºC for 20 min or 37ºC for 30 min and then 50ºC for 20 min) and spotted onto plates to monitor cell viability. (**b**) Hsp26 and Sse1 localize at PACs upon heat shock at 37ºC. Cells expressing Hsp26-GFP and Sse1-GFP were analyzed by fluorescence microscopy after incubation at 37ºC for the indicated time points. (**c**) Scheme depicting possible fates of a temperature-sensitive protein upon heat shock treatment. After temperature upshift misfolded proteins might be sequestered into inclusions (PACs) or targeted to the proteasome for degradation. Once permissive temperature is restored, aggregated proteins can be refolded and recover their functional conformation. Scale bar, 5 μm.

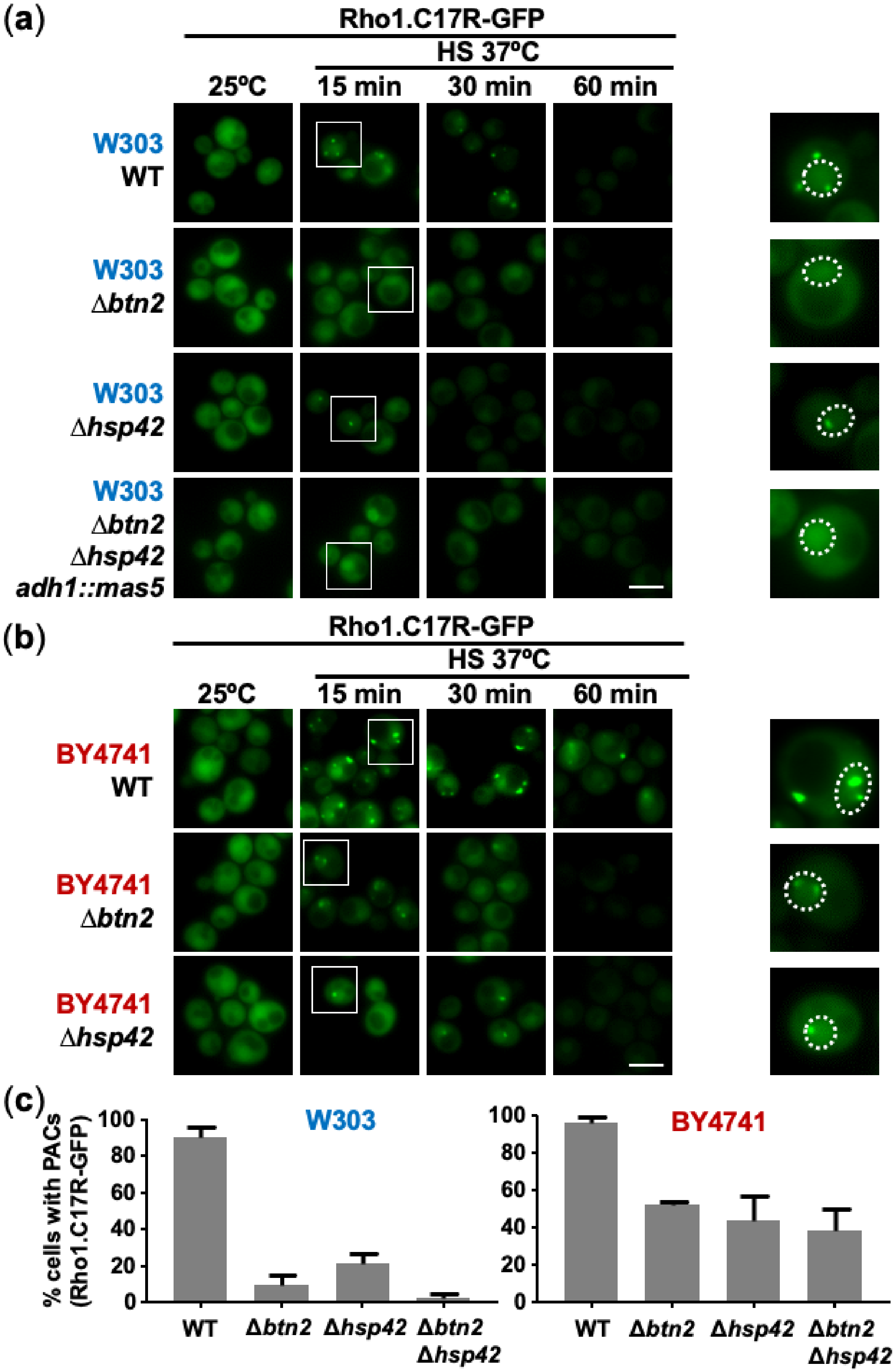

Figure S3. Deletion of Btn2 or Hsp42 have an impact on PAC stability during heat stress. (**a**) Single deletion of Btn2 prevents the assembly of nuclear and cytosolic PACs in W303 strain.

Heat-induced PAC formation was examined by fluorescence imaging in WT, Δ*btn2* and Δ*hsp42* cells. Expression of Mas5 chaperone in cells lacking Btn2 and Hsp42 did not rescue wild-type phenotype. Insets show a magnified region with one cell. Dashed lines indicate the borders of the nuclei. (**b**) Single deletions of Btn2 or Hsp42 do not abolish the assembly of nuclear PACs in BY4741 strain. WT, Δ*btn2* and Δ*hsp42* cells expressing Rho1.C17R-GFP were examined by fluorescence microscopy after incubation at 37ºC for the indicated times. Insets show a magnified region with one cell. Dashed lines indicate the borders of the nuclei. (**c**) PAC formation is compromised in single and double mutants Δ*btn2* and Δ*hsp42.* WT, Δ*btn2,* Δ*hsp4* and Δ*btn2* Δ*hsp42 c*ells expressing Rho1.C17R-GFP were heat-shocked at 37ºC for 15 minutes and the number of cells with PACs from at least 50 cells in each replicate was counted. Graphs represent the average % of cells with PACs from at least three independent experiments. Scale bar, 5 μm.

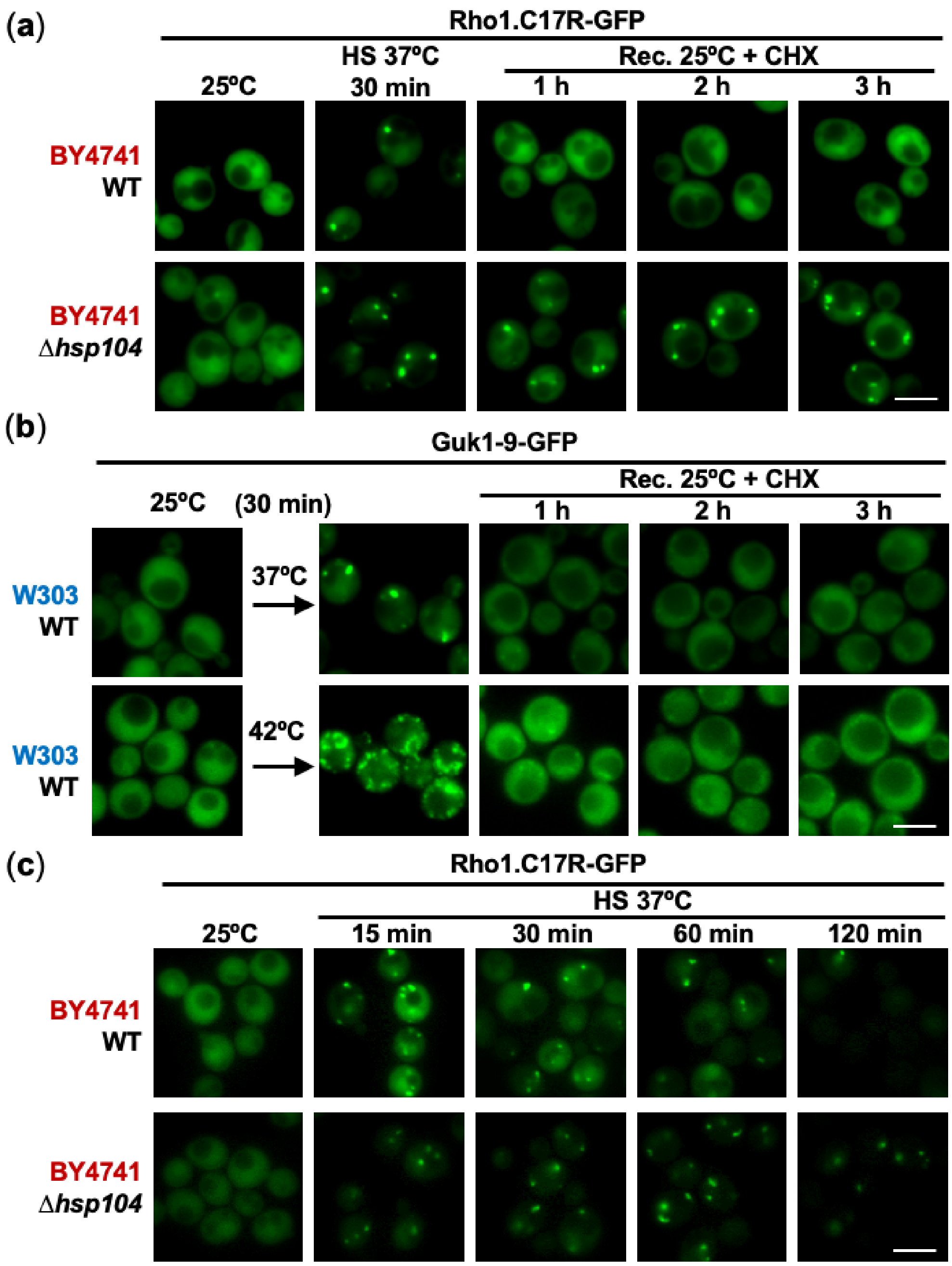

Figure S4. Hsp104 plays a critical role in PAC clearance during heat stress and recovery. (**a**) PACs are disassembled in a Hsp104-dependent manner during stress recovery. Microscopy of WT and Δ*hsp104* cells (BY4741 background) carrying Rho1.C17R-GFP after heat stress at 37°C and recovery at 25°C in the presence of CHX (100 μg/ml). (**b**) PAC disassembly occurs during recovery after severe heat shock. WT cells expressing Guk1-9-GFP were heat-shocked at 37°C for 30 min and 42°C for 15 min and then incubated at 25°C with CHX to block protein synthesis. PAC dissolution during stress recovery was monitored by fluorescence microscopy. (**c**) Hsp104 is also required for PAC disassembly if heat stress persists longer. Stability of PACs labelled with Rho1.C17R-GFP was examined by fluorescence imaging in WT and Δ*hsp104* cells (BY4741 background) after 37°C upshift. Scale bar, 5 μm.

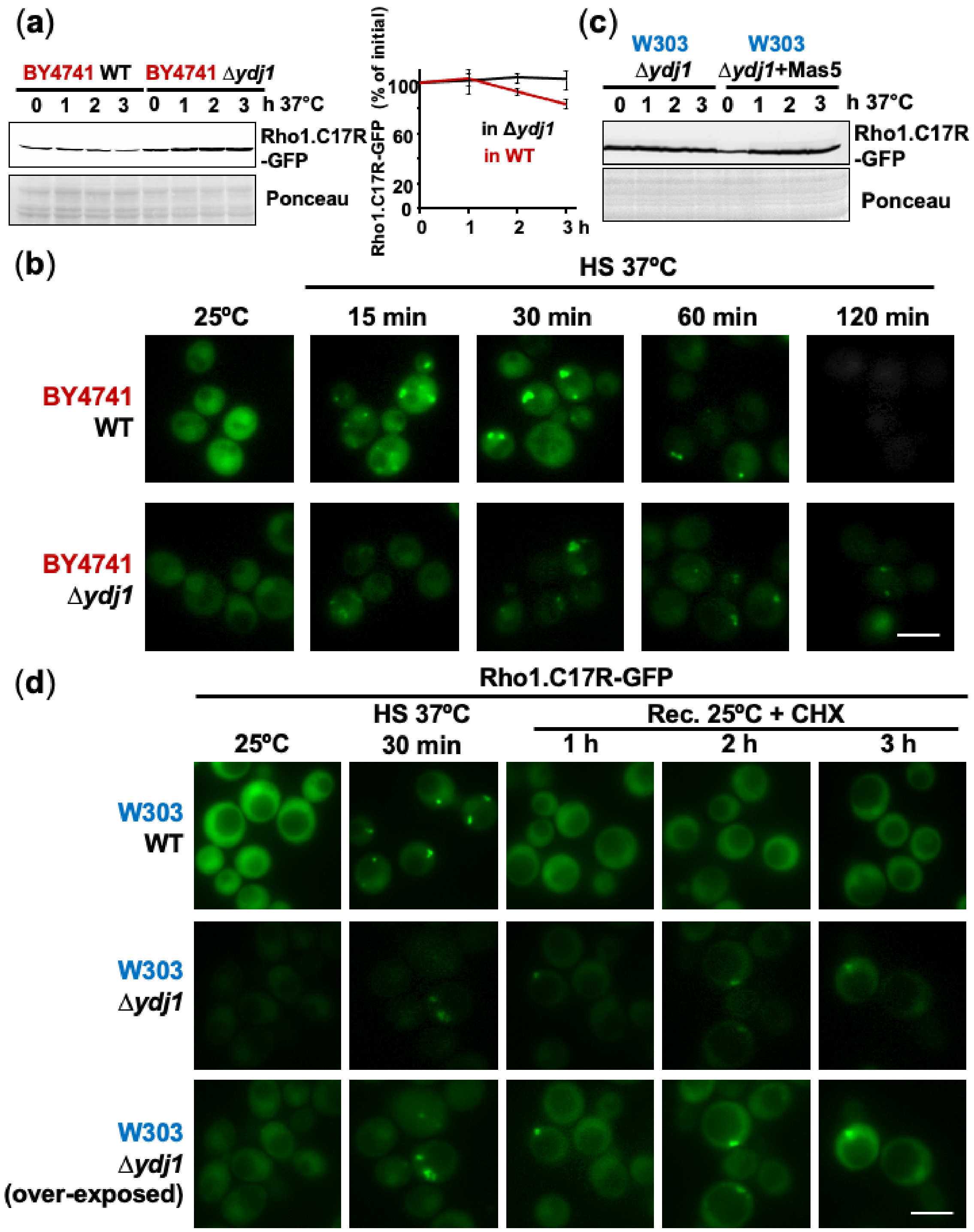

Figure S5. Deletion of the Hsp40 chaperone Ydj1 results in increased PAC and protein stability. (**a**) Protein degradation is not detected in cells lacking Ydj1. Immunoblots showed no change in Rho1.C17R-GFP levels during heat stress at 37°C in Δ*ydj1* strain compared with WT cells. Graph represents the quantification of Rho1.C17R-GFP levels (% of initial) upon 37°C heat shock in WT and Δ*ydj1* cells. Values indicate mean and SD from three independents experiments. (**b**) Heat stress-induced PACs remain longer in Δ*ydj1* cells. PAC formation and stability were examined by fluorescence microscopy in WT and Δ*ydj1* cells (BY4741

background) after incubation at 37ºC for the indicated time points. (**c**) Expression of Mas5 (fission yeast Hsp40) cannot restore protein degradation in Δ*ydj1* cells. Δ*ydj1* and Δ*ydj1 adhi::mas5* cells expressing Rho1.C17R-GFP were heat-shocked at 37ºC for the indicated time periods and the levels of misfolding reporter were detected by immunoblotting using anti-GFP antibody. (**d**) Ydj1 is also critical for PAC clearance during stress recovery. WT and Δ*ydj1* cells were heat-shocked at 37ºC for 30 min and then incubated at 25ºC in the presence of CHX to promote stress recovery. Over-exposure of images is shown to clearly demonstrate the increased PAC stability in Δ*ydj1* cells. Ponceau staining was used as loading control. Scale bar, 5 μm.

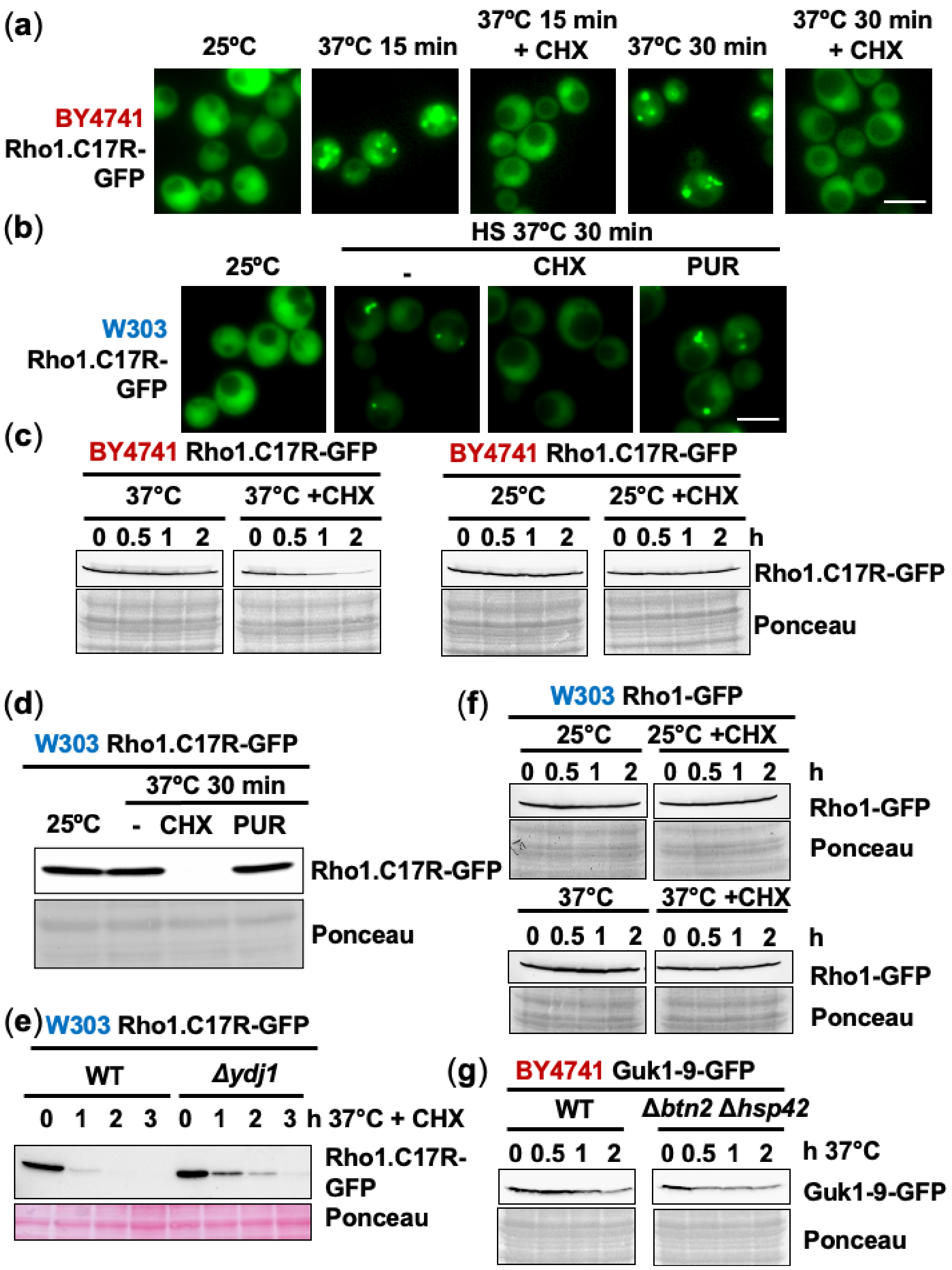

Figure S6. CHX inhibits assembly of PACs and accelerates protein degradation. (**a**) CHX treatment blocks PAC formation under heat stress conditions. Cells (BY4741 background) expressing the reporter Rho1.C17R-GFP were heat-shocked at 37°C for 15 or 30 min in the presence or not of CHX. Assembly of heat-induced PACs was examined by fluorescence microscopy. (**b**) The translation inhibitor puromycin (PUR, 1 mM) did not prevent PAC assembly. Localization of Rho1.C17R-GFP was monitored by fluorescence microscopy after heat stress (37°C, 30 min) imposition. Where indicated, CHX or PUR were used to inhibit protein synthesis. (**c**) Protein degradation is induced when PACs are not assembled.

Immunoblots show enhanced degradation of Rho1.C17R-GFP after heat stress in the presence of CHX, conditions that block PAC formation. Growth at permissive temperature was included to demonstrate that Rho1.C17R-GFP degradation is prompted by protein misfolding. (**d**) CHX, but not PUR, induces protein degradation under heat stress conditions. Rho1.C17R-GFP levels were analyzed by immunoblotting after incubation at 37ºC for 30 min in the presence or not of CHX or PUR. (**e**) Lack of Ydj1 increases protein stability even in the presence of CHX. Rho1.C17R-GFP degradation was analyzed by immunoblotting in WT and Δ*ydj1* cells grown at 37ºC for the indicated time points and in the presence of CHX. (**f**) Rho1 WT is stable upon moderate heat stress. Rho1 WT levels were monitored by immunoblotting after growth at 25ºC or 37ºC in the presence or not of CHX. (**g**) Guk1-9 is degraded more rapidly in cells lacking Btn2 and Hsp42, where PACs disappear earlier. WT and Δ*btn2* Δ*hsp42* cells expressing Guk1-9-GFP were grown at 37ºC for the indicated times and subjected to immunoblot analysis. Ponceau staining was included as loading control. Scale bar, 5 μm.

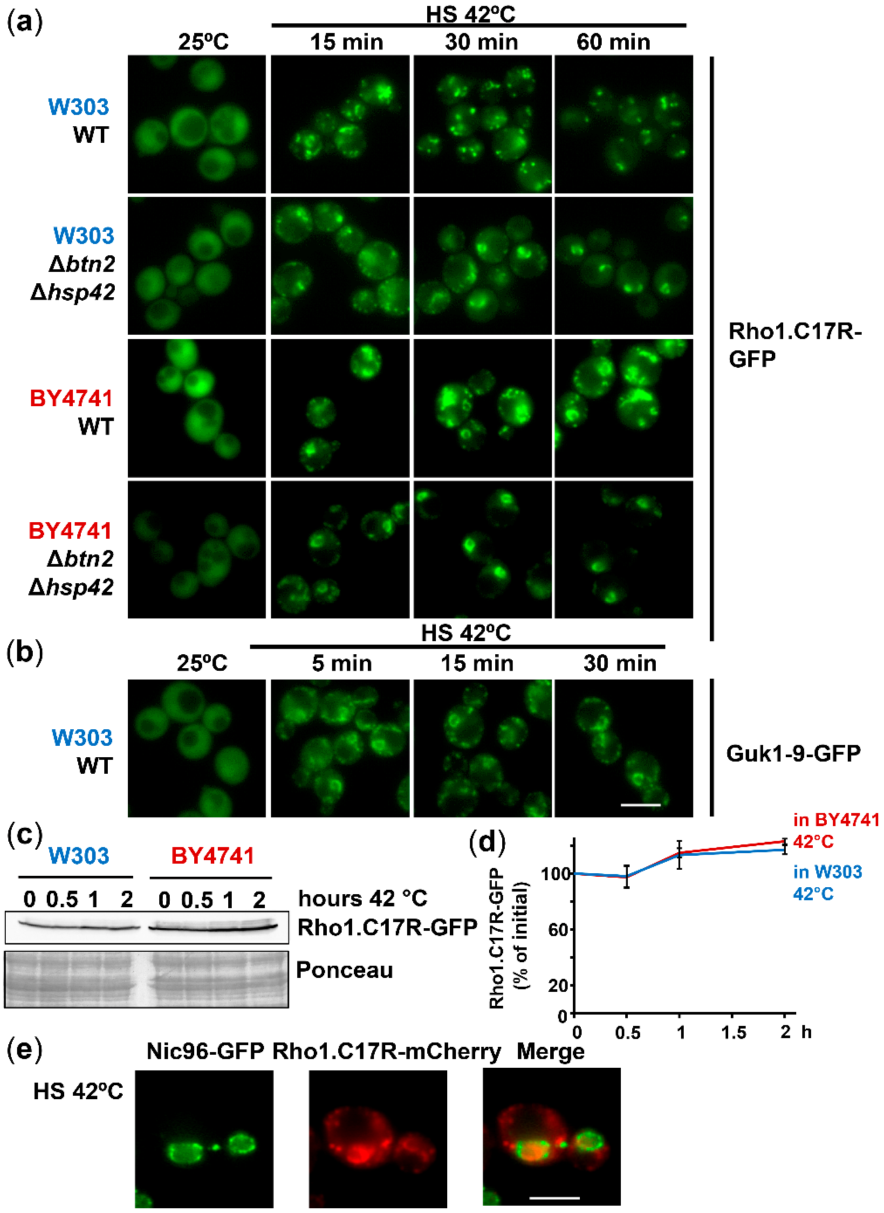

Figure S7. Analysis of protein stability and NuR assembly during severe heat shock. (**a**) Btn2 and Hsp42 are not required for NuRs formation. Rho1.C17R-GFP distribution was monitored by fluorescence microscopy in WT and Δ*btn2* Δ*hsp42* cells after incubation at 42ºC for the indicated time points. (**b**) Guk1-9 localizes at NuRs upon heat stress at 42ºC. Microscopy of cells expressing Guk1-9-GFP exposed to the same stress conditions as in **a**. (**c**) Rho1.C17R is not degraded upon 42ºC upshift. Rho1.C17R levels were detected by immunoblotting after heat shock at 42ºC for the indicated time points. Two strains of budding yeast (W303 and BY4741) were included in this assay. Ponceau staining serves as loading control. (**d**) Quantification of Rho1.C17R levels during heat stress at 42ºC. Strains and growth conditions were the same as in **c** Graph represents the mean value and SD from three independent experiments. **e**) Co-localization of the nucleoporin Nic96 and the NuRs (reporter Rho1.C17R-mCherry) at 42ºC. Scale bar, 5 µm. (

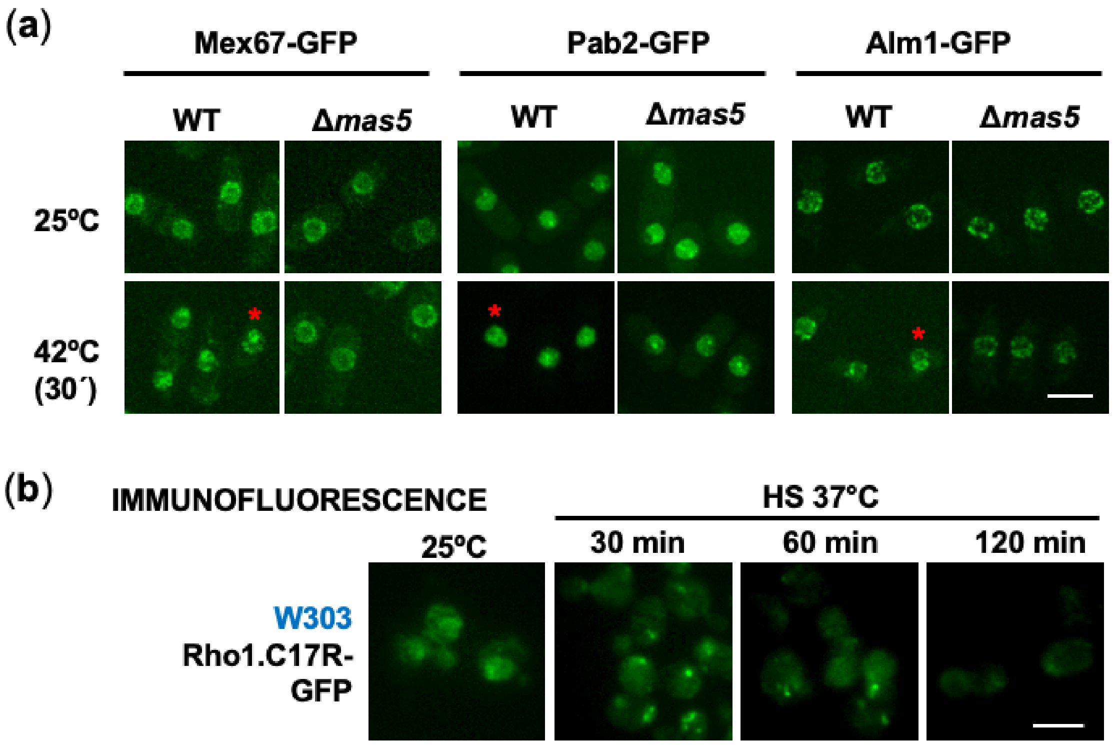

Figure S8. NuR assembly during severe heat shock in *S. pombe* is dependent on Mas5. (**a**) In the absence of Mas5, the RNA binding proteins Mex67-GFP and Pab2-GFP and the nuclear pore complex component Alm1-GFP do not concentrate at NuRs upon heat shock. Cells expressing either Mex67-GFP, Pab2-GFP or Alm1-GFP were grown to mid-log phase and then heat shocked at 42ºC for 30 minutes. Assembly of heat-induced NuRs was examined by fluorescence microscopy. Asterisks mark NuRs. (**b**) The thermo-sensitive mutant Rho1.C17R-GFP concentrates at PACs upon heat shock at 37ºC. Distribution of Rho1.C17R-GFP was analyzed by immuno-fluorescence microscopy at the indicated time points and temperature conditions. Scale bar, 5 μm.

Table S1. Strains used in this study

S. cerevisiae

Strain	Genotype	Origin
BY4741	*MATa his3Δ1 leu2Δ0 met15Δ0 ura3Δ0*	[54]
W303	*MATa leu2-3,112 trp1-1 can1-100 ura3-1 ade2-1 his3-11,15*	[55]
Y01514	*MATa his3Δ1 leu2Δ0 met15Δ0 ura3Δ0 hsp104::kanMX4*	EUROSCARF
Btn2-GFP	*MATa his3Δ1 leu2Δ0 met15Δ0 ura3Δ0 btn2-GFP::HIS3MX6*	GFP collection
Hsp42-GFP	*MATa his3Δ1 leu2Δ0 met15Δ0 ura3Δ0 hsp42-GFP::HIS3MX6*	GFP collection
Hsp104-GFP	*MATa his3Δ1 leu2Δ0 met15Δ0 ura3Δ0 hsp104-GFP::HIS3MX6*	GFP collection
Hsp26-GFP	*MATa his3Δ1 leu2Δ0 met15Δ0 ura3Δ0 hsp26-GFP::HIS3MX6*	GFP collection
Sse1-GFP	*MATa his3Δ1 leu2Δ0 met15Δ0 ura3Δ0 sse1-GFP::HIS3MX6*	GFP collection
MCS299	*MATa leu2-3,112 trp1-1 can1-100 ura3-1 ade2-1 his3-11,15 ADHi::Guk1-9-GFP::HIS3*	This study
MCS308	*MATa leu2-3,112 trp1-1 can1-100 ura3-1 ade2-1 his3-11,15 GPD::Rho1-GFP::HIS3*	This study
MCS309	*MATa leu2-3,112 trp1-1 can1-100 ura3-1 ade2-1 his3-11,15 GPD::Rho1.C17R-GFP::HIS3*	This study
MCS310	*MATa leu2-3,112 trp1-1 can1-100 ura3-1 ade2-1 his3-11,15 GPD::Rho1.C17R-mCherry::HIS3*	This study
MCS311	*MATa leu2-3,112 trp1-1 can1-100 ura3-1 ade2-1 his3-11,15 GPD::Rho1.C17R-GFP::HIS3 ydj1::kanMX6*	This study
MCS312	*MATa his3Δ1 leu2Δ0 met15Δ0 ura3Δ0 GPD::Rho1.C17R-GFP::HIS3*	This study
MCS313	*MATa his3Δ1 leu2Δ0 met15Δ0 ura3Δ0 GPD::Rho1.C17R-GFP::HIS3 hsp104::kanMX4*	This study
MCS319	*MATa his3Δ1 leu2Δ0 met15Δ0 ura3Δ0 htb1-GFP::His3MX6 NOP1::Rho1.C17RmCherry::leu2*	This study
MCS320	*MATa his3Δ1 leu2Δ0 met15Δ0 ura3Δ0 nic96-GFP::His3MX6*<br>*NOP1::Rho1.C17R-mCherry::leu2*	This study
SB685	*MATa leu2-3,112 trp1-1 can1-100 ura3-1 ade2-1 his3-11,15 GPD::Rho1.C17R-GFP::HIS3 ydj1::kanMX6 ADHi::mas5::TRP1*	This study
SB696	*MATa leu2-3,112 trp1-1 can1-100 ura3-1 ade2-1 his3-11,15 GPD::Rho1.C17R-GFP::HIS3 btn2::natMX6*	This study
SB698	*MATa his3Δ1 leu2Δ0 met15Δ0 ura3Δ0 GPD::Rho1.C17R-GFP::HIS3 btn2::natMX6*	This study
SB700	*MATa leu2-3,112 trp1-1 can1-100 ura3-1 ade2-1 GPD::Rho1.C17R-GFP::HIS3 hsp42::kanMX6*	This study

SB702	*MATa his3Δ1 leu2Δ0 met15Δ0 ura3Δ0 GPD::Rho1.C17R-GFP::HIS3 hsp42::kanMX6*	This study
SB703	*MATa leu2-3,112 trp1-1 can1-100 ura3-1 ade2-1 his3-11,15 ADHi::Guk1-9-GFP::HIS3 hsp42::kanMX6 btn2::natMX6*	This study
SB704	*MATa leu2-3,112 trp1-1 can1-100 ura3-1 ade2-1 his3-11,15 GPD::Rho1.C17R-GFP::HIS3 hsp42::kanMX6 btn2::natMX6*	This study
SB705	*MATa his3Δ1 leu2Δ0 met15Δ0 ura3Δ0 GPD::Rho1.C17R-GFP::HIS3 hsp42::kanMX6 btn2::natMX6*	This study
SB720	*MATa his3Δ1 leu2Δ0 met15Δ0 ura3Δ0 GPD::Rho1.C17R-GFP::HIS3 ydj1::hphMX6*	This study
SB734	*MATa his3Δ1 leu2Δ0 met15Δ0 ura3Δ0 ADHi::Guk1-9-GFP::HIS3*	This study
SB736	*MATa leu2-3,112 trp1-1 can1-100 ura3-1 ade2-1 his3-11,15 GPD::Rho1.C17R-GFP::HIS3 hsp42::kanMX6 btn2::natMX6 ADHi::mas5::TRP1*	This study
SB750	*MATa his3Δ1 leu2Δ0 met15Δ0 ura3Δ0 ADHi::Guk1-9-GFP::HIS3 hsp42::kanMX6 btn2::natMX6*	This study
SB756	*MATa leu2-3,112 trp1-1 can1-100 ura3-1 ade2-1 his3-11,15 GPD::Rho1.C17R-GFP::HIS3 hsp104::hphMX6*	This study

S. pombe

**Strain**	**Genotype**	**Origin**
972	*h^-*	[56]
MCS115	*h^- sty1'::guk1-9-GFP::leu1+*	[23]
MCS126	*h^- sty1'::rho1.C17R-GFP::leu1+*	[23]
MV1	*h^- ssa1-GFP::kanMX6*	This study
SB453	*h^- sty1'-GFP-mas5::leu1+*	This study
SB491	*h^- hsp104-GFP::ura4+ ura4D-18*	[23]
SB518	*h^- sty1'-GFP-ssa2::leu1+*	This study
SB557	*h^- hsp104-GFP::ura4+ mas5::kanMX6 ura4D-18*	[23]
RD5954	*h^+ mex67-GFP::kanMX6 leu1-32 ura4-D18*	This study
RD8681	*$h^?$ mas5::kanMX6 mex67-GFP::kanMX6 ura4-D18*	This study
RD7094	*h^- pab2-GFP::kanMX6*	This study
RD8681	*$h^?$ mas5::kanMX6 pab2-GFP::kanMX6 ura4-D18*	This study
RD402	*h^- alm1-GFP::kanMX6 ade6-M216 ura4-D19 leu1-32*	This study
RD8008	*$h^?$ mas5::kanMX6 alm1GFP*	This study

Table S2. Plasmids used in this study

**Plasmid**	**Genotype**	**Origin**
p618’	*sty1’::GFP-mas5::leu1+*	This study
p627’	*sty1’::GFP-ssa2::leu1+*	This study
p714’	*ADHi::Guk1-9-GFP::HIS3*	This study
p740’	*GPD::Rho1-GFP::HIS3*	This study
p741’	*GPD::Rho1.C17R-GFP::HIS3*	This study
p742’	*GPD::Rho1.C17R-mCherry::HIS3*	This study
p769	*NOP1::Rho1.C17R-mCherry, 2 micron.*	This study
p810’	*ADHi::mas5::TRP1*	This study

REFERENCES

23. Cabrera, M.; Boronat, S.; Marte, L.; Vega, M.; Perez, P.; Ayte, J.; Hidalgo, E. Chaperone-Facilitated Aggregation of Thermo-Sensitive Proteins Shields Them from Degradation during Heat Stress. Cell Rep. 2020, 30, 2430–2443.e4.

54. Baker Brachmann, C.; Davies, A.; Cost, G.J.; Caputo, E.; Li, J.; Hieter, P.; Boeke, J.D. Designer deletion strains derived from Saccharomyces cerevisiae S288C: a useful set of strains and plasmids for PCR-mediated gene disruption and other applications. Yeast 1998, 14, 115–132.

55. Rothstein, R.J. One-step gene disruption in yeast. Methods Enzym. 1983, 101, 202–211.

56. Leupold, U. Genetical methods for Schizosaccharomyces pombe. Methods Cell Physiol. 1970, 4, 169–177.
